# Awareness of Shaken Baby Syndrome among Saudi Nursing Students: A Cross-Sectional Study

**DOI:** 10.3390/healthcare12121203

**Published:** 2024-06-15

**Authors:** Amany Anwar Saeed Alabdullah, Hala Kadry Ibrahim, Raneem Nezar Aljabal, Ahad Mohammed Awaji, Bayan Abdullah Al-otaibi, Fay Meshal Al-enezi, Ghada Saud Al-qahtani, Hawazen Hassan Al-shahrani, Raneem Saleem Al-mutairi

**Affiliations:** 1 Department of Maternity and Pediatric Nursing, College of Nursing, Princess Nourah bint Abdulrahman University, P.O. Box 84428, Riyadh 11671, Saudi Arabia; 2 Community, Psychiatric and Mental Health Nursing Department, College of Nursing, Princess Nourah bint Abdulrahman University, P.O. Box 84428, Riyadh 11671, Saudi Arabia; hkabdelfattah@pnu.edu.sa; 3 College of Nursing, Princess Nourah bint Abdulrahman University, P.O. Box 84428, Riyadh 11671, Saudi Arabia; 441003861@pnu.edu.sa (R.N.A.); 441002860@pnu.edu.sa (A.M.A.); 441002883@pnu.edu.sa (B.A.A.-o.); 441004009@pnu.edu.sa (F.M.A.-e.); 441003063@pnu.edu.sa (G.S.A.-q.); 441004576@pnu.edu.sa (H.H.A.-s.); 441001854@pnu.edu.sa (R.S.A.-m.)

**Keywords:** shaken baby syndrome, nursing, knowledge, nursing curricula, abusive head trauma, non-accidental head injury

## Abstract

Child abuse is a global problem. Shaken baby syndrome (SBS) is a result of child abuse, with shaking being the most common form of maltreatment, causing mortality or severe brain damage in infants. A lack of awareness of SBS among current and future healthcare professionals can have serious consequences. To date, no studies have been conducted in Saudi Arabia to examine student nurses’ awareness of SBS, so we sought to assess this issue in an academic institution in Riyadh, Saudi Arabia. For this questionnaire-based study, we employed a cross-sectional, descriptive design. The target population was nursing students from every year of study in the institution’s five-year undergraduate nursing programme, who received an online questionnaire during the 2022–2023 academic year. The data were analysed using descriptive and inferential statistical analysis. Of the 293 respondents, 100.0% confirmed that they were not aware of SBS through their nursing curricula, and 62.1% reported not being made aware of SBS at all during their academic journey. The majority of participants were unaware of the negative consequences of shaking a baby. Most were not aware that shaking a baby vigorously can cause permanent blindness (73.4%), postural impairments (56.7%), sleep disorders (61.1%), or convulsions (60.1%). The results of our study revealed a statistically significant relationship between nursing students’ awareness of SBS and both their year of study and marital status. Those at higher academic levels and those who were married were more aware of SBS. To improve nursing students’ knowledge of SBS and help them to better inform the public of this syndrome, particularly parents, child maltreatment topics should be added to nursing curricula in Saudi Arabia, and their importance should be emphasised. This will help reduce the prevalence and burden of SBS nationally.

## 1. Introduction

Child maltreatment involves the abuse or neglect of children under 18 years of age and can take many forms, including physical, emotional, or sexual abuse; physical or emotional neglect; or exploitation by a caregiver [1,2]. Child abuse is defined as “an act or failure to act on the part of a parent or caretaker that results in death, serious physical or emotional harm, sexual abuse, or exploitation; or an act or failure to act which introduces an avoidable danger or substantial damage to anyone under the age of 18” [3]. Physical child abuse involves the intentional use of any physical force against a child that results (or is highly likely to result) in harm or injury to the child’s health or development. Physical abuse can take many forms, including “beating, shaking, kicking, burning, biting, suffocating, or poisoning, regardless of the perpetrator’s intention” [4,5].

Shaken baby syndrome (SBS), also referred to as abusive head trauma [6] or non-accidental head injury [7], occurs as a result of child abuse, specifically physical maltreatment. It may occur when a parent or other adult handling a baby becomes angry, loses control, and shakes the baby, often because the child will not stop crying [8]; however, head injuries in infants may result from a range of deliberate actions [9]. For the purposes of this article, we use the term SBS, defined as “a devastating form of inflicted traumatic brain injury that occurs when a young child is violently shaken and subjected to rapid acceleration, deceleration and rotational forces, with or without impact” [10]. SBS is the most common cause of mortality or severe brain damage from maltreatment in infants [11,12]. The syndrome is unique to childhood because infants have weak neck muscles and are unable to maintain the position of their relatively large heads. As a consequence, the extreme vibration associated with shaking causes repeated back-and-forth movement of the infant’s head, which can significantly or even fatally damage the brain. If the infant’s head strikes a surface during shaking, the risk of harm is amplified [13]. There is no pattern of cranial injury that is specifically diagnostic of SBS, although retinal haemorrhages and subdural haemorrhages in certain or multiple locations are more common with SBS than with accidental injury [6].

It is important to note that parenting norms differ from country to country [2]. For instance, in Jordan, an Arab country, 68.0% of parents reported that, when they were anxious or fatigued, they shook their children to calm them down [14]. Similarly, Alanazi [15] and Al Dosari et al. [16] reported that, in Saudi Arabia, a considerable and alarming percentage of parents had misconceptions regarding their right to hit their children to stop them from being noisy. In another study in Saudi Arabia, 24.0% of parents reported forcefully shaking their infants, while 41.0% described throwing their infants into the air and catching them [17]. Another study in Saudi Arabia found that 58.0% of parents reported shaking their infants to calm them down [18]. Similarly, Al Omran et al. [19] reported that 80.0% of parents in Saudi Arabia shook their infants to calm them when they were crying and 14.0% shook their infants when playing with them. Other studies conducted in Saudi Arabia have found that parents have limited awareness of child abuse in general [16,20] and SBS in particular [17,18,19,21,22].

Al Omran et al. [19] focused on SBS as a form of child abuse and assessed parents’ levels of knowledge and awareness of SBS. The majority of parents (68.0%) were unaware of SBS, and 25.0% thought that violently shaking a child would have no consequences. Alshahrani and colleagues [18] reported that 70.0% of their respondents had never heard of SBS, and 67.0% were unaware of the risks associated with shaking an infant and the relationship between shaking infants and causing them harm. Felemban et al. [22] found that 65.0% of parents in Saudi Arabia had never heard of or read about SBS and 71.0% did not know the cause of SBS. Alzahrani et al. [17] determined that 69.0% of parents surveyed were not aware of SBS.

These results highlight parents’ lack of knowledge of SBS and the corresponding need for education. Alreshidi et al. [21] explored public perspectives on different types of child abuse and found that people were only moderately aware that violent shaking constitutes abuse. These results show that some forms of child abuse, including SBS, might not be sufficiently highlighted in awareness campaigns and thus require more coverage; increasing parents’ awareness could reduce the incidence of SBS and related complications [23]. Alreshidi et al. [21] found that 35.0% of respondents thought the best approach to protecting a child from abuse was to educate families about the issue. Alzahrani et al. [17] found that 84.0% of parents had a positive attitude towards learning more about SBS, while 40.0% and 34.0% were interested in learning more about SBS before and during pregnancy, respectively.

One of the goals of the Child Protection Strategy of the United Nations International Children’s Emergency Fund (UNICEF) is to improve the knowledge, motivation, and support of those in contact with children, including nurses, to better protect children from harm [24]. Given the value of sharing related knowledge with both the community and caregivers, it is essential that nurses’ education prepares them to do so. Nurses are at the frontline of patient care and play an important role in health promotion and prevention; one way they can play that role is via health education [25].

Nursing students have ongoing relationships with patients, families, and caregivers at all levels of healthcare. They play an important role in providing care and raising public awareness of health-related issues, so knowledge of SBS and its negative consequences is crucial. Nursing students must have sufficient awareness, a clear understanding, and up-to-date information about SBS and its associated risks. Therefore, in the present study, we sought to assess nursing students’ awareness of SBS.

To the best of our knowledge, no prior studies have examined the content and relevance of undergraduate nursing education in Saudi Arabia with regard to child maltreatment, particularly SBS. Therefore, national data about SBS awareness among undergraduate nurses are unavailable. We anticipate that the results of our study will aid in planning interventions to improve awareness of SBS among nursing students.

## 2. Materials and Methods

We employed a cross-sectional, descriptive design. The study setting was a college of nursing at an all-female academic institution in Riyadh, Saudi Arabia, with a total of 1219 students. The Raosoft sample size calculator [26] was used to determine the sample size needed for the study (*n* = 293) (Raosoft, 2004). The margin of error was kept at 5%, and the confidence level for the study was 95%. The sample included nursing students from each year of study, in proportion with the total number of students in each year of the undergraduate nursing programme (first-, second-, third-, and fourth-years and interns), during the 2022–2023 academic year to investigate the topic of research from different perspectives (see Supplementary Table S1).

Data were gathered from 293 eligible nursing students who were willing to participate and did not withdraw from the programme during the data collection period. With the support of academic affairs, an email with a link to the questionnaire and information about the study was sent to all students in the college. The exclusion criteria included students who did not approve of participating. Data were collected from April to May 2023 using a modified questionnaire developed by Eyisi et al. [27] after obtaining the authors’ approval to modify the instrument. The original questionnaire consisted of two sections: knowledge of SBS and practice of SBS. This modified and validated tool was used after omitting the practice questions; only the awareness questions were included, as they covered the purpose and aim of the study appropriately, which was to assess nursing students’ awareness of SBS. The questions were appropriate for the study population’s level of knowledge and were culturally appropriate for Saudi Arabia. The questionnaire was written in English and structured in two parts. The first part gathered data on the respondents’ demographic characteristics (age, year of study, marital status, and number of children), and the second part gathered data on their awareness of SBS. The second part included nine items with options of ‘aware’ and ‘not aware’, with one question asking whether the students were taught about SBS in the curriculum of their undergraduate nursing programme. The content of the questionnaire was validated and reviewed by a panel of five experts: one in maternal nursing, two in community nursing, one in paediatric nursing, and one paediatrician expert. All were research experts and confirmed that the questionnaire was suitable, with no modifications required. A pilot study was conducted using a purposive sample of 10 nursing students to determine the questionnaire’s validity and ensure its clarity and accuracy. The pilot study participants were excluded from the main study sample. Cronbach’s alpha coefficient test was employed to evaluate the tool’s internal consistency and evaluate its reliability (=0.86 at *p* < 0.05).

The final version of the questionnaire was uploaded to Google Forms and distributed to the nursing students online so that they could answer in their free time in a suitable place. This was important, considering the load of their programme, which included theory classes and clinical training days. It took the respondents an average of five minutes each to complete the questionnaire.

This study was approved by the academic institutional review board (approval number HAP-01-R-059, 9 April 2023). Respondents’ informed consent to participate was obtained by asking them to check the first box of the questionnaire prior to answering the questions. The respondents were informed that they could withdraw from the study at any time for any reason, and that their participation was entirely voluntary. Great care was taken to ensure the confidentiality of the participants’ data, and only the primary researcher had access to the data on a password-protected personal computer.

### Statistical Analysis

Both descriptive and inferential statistics were derived from the collected data using IBM SPSS Statistics (Version 23). The demographic data were summarised, organised, and simplified using descriptive statistics, including frequencies and percentages. The chi-square test was used to assess the relationship between demographics and awareness of SBS among nursing students. A *p*-value of <0.05 was considered to be statistically significant.

## 3. Results

### 3.1. Descriptive Statistics

#### 3.1.1. Respondents’ Demographic Profile

Table 1 summarises the demographic characteristics of the nursing students who participated in this study. The response rate was 95.0%. The majority of nursing students (75.8%) were 19 to 21 years old. Most (47.4%) were in the first year of their nursing education. The majority (95.6%) were single, and nearly all (98.0%) had no children.

#### 3.1.2. Awareness of SBS among Nursing Students

Table 2 summarises the level of awareness of SBS among the nursing students who participated in this study. All students reported that they had no prior exposure to any curricula relating to or mentioning SBS. Most of the nursing students (62.1%) indicated that they were unaware of SBS. A substantial proportion (74.7%) of the nursing students recognised that shaking an infant vigorously can cause serious harm, and 54.6% acknowledged that an infant could potentially die as a result of severe shaking. However, there were notable deficiencies in the nursing students’ knowledge of the specific consequences of shaking a baby. For instance, 73.4% of the nursing students were unaware that vigorous shaking could lead to permanent blindness, while 43.3% did not realise that it could impair an infant’s posture.

### 3.2. Inferential Statistics

Chi-square analyses were used to investigate associations between awareness of SBS and the nursing students’ demographic characteristics, the findings of which are presented in Table 3. The majority of the participants lacked knowledge of SBS in all age groups except for those older than 27, although the sample size of those participants was very small (*n* = 3). There was no statistically significant association between age and awareness of SBS among nursing students χ^2^ (3, *n* = 293) = 4.644, *p* > 0.001, V = 0.122.

A chi-square analysis was employed to explore the association between awareness of SBS and the nursing students’ year of study. The majority of nursing students in their first or second year of study were unaware of SBS, while the opposite was true for third- and fourth-year students (although most interns were unaware of SBS) (Table 3). The chi-square test yielded a statistically significant result χ^2^ (1, *n* = 293) = 15.358, *p* < 0.001, V = 0.299, indicating a significant association between the nursing students’ year of study and their awareness of SBS. Moreover, the Cramer’s V coefficient of 0.229 suggested a moderate effect size, underscoring the strength of the identified association.

With regard to marital status, the chi-square test showed a statistically significant association between the nursing students’ marital status and their awareness of SBS χ^2^ (1, *n* = 293) = 8.038, *p* < 0.001, V = 0.164; nursing students who were married were more likely to be aware of SBS. Additionally, the Cramer’s V coefficient of 0.164 indicated a moderate effect size, emphasising the strength of the observed association (Table 3). There was no statistically significant association between the nursing students’ number of children and their awareness of SBS χ^2^ (2, *n* = 293) = 5.765, *p* > 0.001, V = 0.137. The Cramer’s V coefficient of 0.137 indicated a small effect size, further supporting the lack of a substantial association.

## 4. Discussion

SBS is a major problem that can affect neonates and infants worldwide. It may be even more widespread than it initially appeared to be, with its prevalence highly underestimated [13]. In Saudi Arabia, child abuse and neglect, including SBS, are under-reported [28], even though paediatricians may regularly encounter such cases. Nursing students play an important role in educating the public. As future nurses, they must be sufficiently prepared with the necessary information. Awareness of the different types of child abuse is crucial to dealing with associated problems [29,30,31]. Thus, nursing curricula should integrate SBS into well-prepared courses that engage students at an early stage to ensure that they can educate caregivers about preventive measures. Our findings demonstrate that there is currently insufficient awareness of SBS among this cohort, and this knowledge has not been taught to them or included in the curriculum.

A study in Iraq by Shaker and colleagues [32] involving 50 nurses from various maternity and paediatric units found that most nurses (88.0%) had poor knowledge of SBS. This is in line with our finding that 62.1% of nursing students were unaware of SBS. Only 25.0% of the nurse respondents in Shaker et al.’s study knew that brain damage, cerebral palsy, and blindness are all complications of SBS, which is similar to our finding that only 26.6% of nursing students knew that shaking a neonate vigorously can cause permanent blindness. This is also consistent with the results of Al Omran et al. [19], who reported that only 33.6% of parent participants recognised that shaking was a form of abuse capable of causing harmful serious health issues; only 11.3% knew that shaking could cause blindness, and 71.2% did not know that shaking a neonate could lead to death. We found that 45.4% of nursing students were unaware that SBS could cause death. These findings indicate that parents and nursing students alike have poor awareness and knowledge of SBS.

The results of our study revealed a statistically significant relationship between nursing students’ awareness of SBS and both their year of study and marital status. Specifically, the results showed that the clinical-year nursing students (i.e., those in the third and fourth years of their nursing programme or in their internship) were more aware of SBS than preclinical-year nursing students (those in the first or second year). The clinical years include paediatric and maternity nursing in the third year of the programme and community nursing in the fourth year, and students have contact with children and families. Clinical-year nursing students were more likely to have encountered relevant patients, which could explain why more of them had insight into SBS. Another reason might be their more up-to-date knowledge, continuous reading, and active participation in awareness campaigns, in line with the findings of Al-Qahtani et al. [29]. We also found that there was an association between marital status and level of awareness of SBS, as married respondents were more aware of SBS than either single or divorced respondents. On the other hand, no significant association was observed for age or number of children, which is in agreement with the findings of Al Omran et al. [19].

Conducting research into the prevalence of SBS in Saudi Arabia is essential for assessing the scale of the problem and ensuring that the wider Saudi society has sufficient awareness of the syndrome to take active measures to address it. Given that prevention is better than cure, Saudi health authorities should increase their efforts to raise public awareness of SBS, including among nursing students. This would represent an essential step towards reducing the prevalence of SBS and its consequences. If public awareness of this problem increases, the practice of shaking neonates or infants in an attempt to modify their behaviour will be increasingly condemned by society. Awareness campaigns should address the misconceptions of both nursing students and members of the public so that they recognise SBS practices as abusive, which should help to prevent such practices.

Our findings highlight the importance of increasing the awareness of SBS among nursing students. This could be achieved by including the topic of child maltreatment in nursing curricula. Early inclusion of SBS at the beginning of nursing programmes could be achieved by requiring students to attend conferences and seminars that cover the topic. Its consequences should be highlighted in awareness programmes, seminars, and lectures. As nursing students come into regular contact with paediatric patients and their families from the start of their nursing education programmes, they are in an excellent position to provide health education to families or caregivers. They also participate in many community services that could be used to spread knowledge and raise public awareness of SBS.

The results of our study can be used as baseline data for evaluating the effectiveness of future interventions intended to improve nursing students’ knowledge of SBS and help them better inform the public, particularly parents. A partnership between undergraduate nursing students and obstetrics to provide an educational programme to 148 pregnant and postpartum mothers about coping with infant crying and the dangers of shaking a baby showed a significant effect on mothers’ knowledge of SBS and coping strategies for crying, while students also developed in their roles as educators and researchers [33]. It is essential to reinforce nursing students’ knowledge to help reduce the incidence and burden of this condition.

As the issue of SBS is important and relevant both nationally and globally, we recommend that further studies of this type be conducted throughout nursing colleges in Saudi Arabia to identify the needs and knowledge gaps of nursing students. Such studies will help college deans and faculties to develop more comprehensive and effective nursing curricula.

### 4.1. Strengths

The present study extends the literature on nursing students’ awareness of SBS and highlights the importance of improving education regarding this crucial topic during undergraduate studies. To the best of our knowledge, no similar studies have previously been conducted among nursing students in Saudi Arabia. A few studies have examined medical students’ awareness of child abuse, but not specifically SBS [23,29,34].

### 4.2. Limitations

Our study was conducted at just one nursing college for female nursing students in Saudi Arabia, and it would be interesting to extend this study to all nursing colleges as part of a country-wide investigation. A larger sample size and the inclusion of varied participants (including male nursing students) from all nursing programmes in Saudi Arabia would provide a better overview of the topic at hand. Despite these limitations, the present study included a relatively large number of participants, which helped to counteract potential bias. Other limitations concern the use of an online survey for data collection, which poses a high risk of receiving fraudulent answers and biased responses.

## 5. Conclusions

In this study, we examined the associations between nursing students’ awareness of SBS and various demographic factors, revealing nuanced patterns. A significant association was found between nursing students’ academic progression and their awareness of SBS. Marital status also played a role, and our analysis indicated that married nursing students exhibited greater awareness of SBS than their divorced or single counterparts. In contrast, the age of the nursing students or the number of children they had did not significantly influence nursing students’ awareness of SBS.

## Figures and Tables

**Table 1:** Demographic characteristics of nursing students who participated in this study.

Demographic Characteristics	Frequency	Percent
Age (years)	(*n* = 293)	
19–21	222	75.8%
22–24	67	22.9%
25–27	1	0.3%
>27	3	1.0%
Year of study	(*n* = 293)	
First year	139	47.4%
Second year	55	18.8%
Third year	32	10.9%
Fourth year	31	10.6%
Internship year	36	12.3%
Marital status	(*n* = 293)	
Divorced	1	0.3%
Married	12	4.1%
Single	280	95.6%
Number of children	(*n* = 293)	
No children	287	98.0%
1–2 children	5	1.7%
3–5 children	1	0.3%

**Table 2:** Awareness of SBS among nursing students who participated in the study.

	Frequency	Percent
Aware of SBS through curricula		
No	293	100.0%
Yes	0	0.0%
Aware of SBS in general		
No	182	62.1%
Yes	111	37.9%
Shaking a baby vigorously can cause serious harm		
No	74	25.3%
Yes	219	74.7%
A baby can die as a result of severe shaking		
No	133	45.4%
Yes	160	54.6%
Shaking a baby vigorously can cause permanent blindness		
No	215	73.4%
Yes	78	26.6%
Shaking a baby vigorously can cause postural impairments		
No	166	56.7%
Yes	127	43.3%
Shaking a baby vigorously can result in sleep disorders		
No	179	61.1%
Yes	114	38.9%
Shaking a baby vigorously can cause bone fractures		
No	144	49.1%
Yes	149	50.9%
Shaking a baby vigorously can cause convulsions		
No	176	60.1%
Yes	117	39.9%

**Table 3:** Associations between nursing students’ awareness of SBS and their demographic characteristics.

Demographic Characteristics	Aware of SBS		χ^2^	*p*-Value	Cramer’s V
	No	Yes			
	(*n* = 182)	(*n* = 111)			
Age (Years)			4.644	0.223	0.122
19–21	144	78			
	64.9%	35.1%			
22–24	36	31			
	53.7%	46.3%			
25–27	1	0			
	100.0%	0.0%			
>27	1	2			
	33.3%	66.7%			
Year of study			15.358	0.004 *	0.229
First year	98	41			
	70.5%	29.5%			
Second year	36	19			
	65.5%	34.5%			
Third year	12	20			
	37.5%	62.5%			
Fourth year	15	16			
	48.4%	51.6%			
Internship year	21	15			
	58.3%	41.7%			
Marital status			8.038	0.018 *	0.164
Divorced	1	0			
	100.0%	0.0%			
Married	3	9			
	25.0%	75.0%			
Single	178	102			
	63.6%	36.4%			
Number of children			5.765	0.056	0.137
No children	181	106			
	63.1%	36.9%			
1–2 children	1	4			
	20.0%	80.0%			
3–5 children	0	1			
	0.0%	100.0%			

* Significant at the *p* < 0.05 level.

## Data Availability

The data presented in this study are only available on request from the corresponding author for reasons of confidentiality.

## Supplementary Materials

The following supporting information can be downloaded at: https://www.mdpi.com/article/10.3390/healthcare12121203/s1, Supplementary Table S1: Total population and study sample size.
