# Supplementary material for: Awareness of Shaken Baby Syndrome among Saudi Nursing Students: A Cross-Sectional Study
Source: Healthcare (Basel). 2024 Jun 15;12(12):1203. doi: 10.3390/healthcare12121203 (PMC11202830; doi:10.3390/healthcare12121203)
Supplement: Supplementary file 1 [file healthcare-12-01203-s001.zip › healthcare-3010981-supplementary.pdf]

Supplementary Table S1: Total population and study sample size.

**College**	**No. of student**	**Formula**
**Nursing**	**N=1219**	**n= 293**

Sampling calculation based on (95% Confidence, 5% error)

**Year of study**	**Number of students**	**Calculated population**
First year of nursing education	578	139
Second year of nursing education	229	55
Third year of nursing education	134	32
Fourth year of nursing education	129	31
Internship year	149	36
